# Promoting a Hand Hygiene Program Using Social Media: An Observational Study

**DOI:** 10.2196/publichealth.5101

**Published:** 2016-02-02

**Authors:** Sung-Ching Pan, Wang-Huei Sheng, Kuei-Lien Tien, Kuang-Tse Chien, Yee-Chun Chen, Shawn-Chwen Chang

**Affiliations:** ^1^ National Taiwan University Hospital Taipei Taiwan

**Keywords:** social media, hand hygiene, infection control

## Abstract

**Background:**

Hand hygiene is an important component in infection control to protect patient safety and reduce health care-associated infection.

**Objective:**

Our aim was to evaluate the efficacy of different social media on the promotion of a hand hygiene (HH) program.

**Methods:**

The observational study was conducted from May 5 to December 31, 2014, at a 2600-bed tertiary care hospital. A 3-minute video of an HH campaign in 8 languages was posted to YouTube. The Chinese version was promoted through three platforms: the hospital website, the hospital group email, and the Facebook site of a well-known Internet illustrator. The video traffic was analyzed via Google Analytics. HH compliance was measured in November 2013 and 2014.

**Results:**

There were 5252 views of the video, mainly of the Chinese-language version (3509/5252, 66.81%). The NTUH website had 24,000 subscribers, and 151 of them viewed the video (connection rate was 151/24,000, 0.63%). There were 9967 users of the hospital email group and the connection rate was 0.91% (91/9967). The connection rate was 6.17% (807/13,080) from Facebook, significantly higher than the other 2 venues (both *P*<.001). HH compliance sustained from 83.7% (473/565) in 2013 to 86.7% (589/679) in 2014 (*P*=.13) among all HCWs.

**Conclusions:**

Facebook had the highest connection rate in the HH video campaign. The use of novel social media such as Facebook should be considered for future programs that promote hand hygiene and other healthy behaviors.

## Introduction

Since the World Health Organization (WHO) launched the first global hand hygiene (HH) campaign to protect patient safety in 2005, more than 130 WHO member states and 17,000 health care facilities have committed to improving HH [1]. Traditionally, printed posters were an important component in the multimodal HH-promoting strategies and other healthy behaviors in the work place [2]. However, there is typically no systemic evaluation to quantify how many people viewed printed posters, and changes in HH compliance may be due to the poster or other multimodal strategies to improve HH [3].

We have therefore questioned whether novel methods can be used to engage our health care workers (HCWs) and accurately measure the efficacy of an HH promotion. “Net generation” has been used to describe the generation who grew up in the era of electronic devices and social media [4]. A 2012 report from the Institute for Information Industry indicated that 74.5% of the population in Taiwan uses the Internet [5]. Thus, in addition to poster reminders, we aimed to promote an HH program in our institution via social media.

In Taiwan, the WHO’s HH program was centrally sponsored by the Centers for Disease Control (CDC) of Taiwan from 2009-2011 in three demonstration centers. After that, the program was distributed throughout the nation via HH certification programs [6]. In 2014, the National Taiwan University Hospital (NTUH) highlighted the HH program through a video campaign to remind our HCWs about the importance of HH and was launched on world HH day (May 5, 2014). Three different platforms were used to promote the video [7]: the hospital website, the hospital group email system, and a well-known Facebook site owned by a medical school student of the National Taiwan University (NTU). We compared the efficacy of promoting the HH program on these three different platforms.

## Methods

### Ethics Statement

This study was approved by the ethics committee of the NTUH. Informed consent was waived under the agreement of the committee (201501083W).

### Setting

The NTUH (Taipei, Taiwan) is a university-affiliated medical center with 2600 beds. Alcohol-based hand hygiene equipment has been provided hospital-wide since 2004 [8]. A centrally sponsored HH program was conducted from 2009-2011.

### Hand Hygiene Video

The video script was written by one of the authors (PSC) and produced by the FTIG Company (Taipei, Taiwan). The main actors were HCWs in the NTUH. Eight versions of the videos were produced in different languages, including the most common languages in Taiwan (Chinese, Hokkien, and Hakka) and languages commonly used by international patients (English, Japanese, French, Indonesian, and Vietnamese).

The 3-minute video covered three main topics: an introduction to the importance of HH and the commitment of the NTUH, a short drama to demonstrate five different moments for HH, and a dancing section that demonstrated the technique of HH.

### Video Broadcast

The videos were uploaded to YouTube on the official site of the Center for Infection Control, NTUH. The videos can be found by searching for keywords such as “National Taiwan University Hospital (NTUH)” and “hand hygiene.” The videos were launched on May 5, 2014. The Chinese version was promoted through three sites: (1) the website of NTUH via an article introducing World HH day, (2) NTUH group email, and (3) the well-known Facebook site of a 6^th^ year medical student at the NTU [7]. This student’s Facebook group, “Clerk: the life as a road blocker,” was started in 2013 and shared the life of a clerk who rotated in the training hospital. The video was shared with a comic who demonstrated the importance of HH on May 5, 2014.

### Hand Hygiene Compliance

The method used to determine HH compliance was in accordance with WHO [2]. Covert observation were conducted by trained infection control nurses (ICNs). The HH observation was around 20 minutes in every ward. At least 6 HCWs, including physicians and nurses, were randomly selected. The ICNs would introduce themselves before the observation and give brief feedback right after the observation was completed. The compliance rate was calculated by the number of actual HH actions, divided by the number of required HH actions. The hospital-wide HH compliance rates were gathered and feedback from the chief and head nurse of every ward. HH compliance was checked annually in November, and the compliance rates in 2013 and 2014 were compared.

### Statistics

The number of website views was determined by Google Analytics. The connection rate was calculated by the number of video views divided by the users subscribed to the specific platforms and compared by chi square test. A *P* value ˂.05 was defined as statistically significant. The statistical analyses were performed using STATA 11.0.

## Results

**Figure 1:**
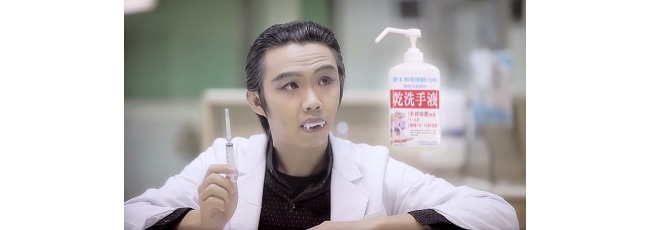
Screenshot of the video “5 moments for hand hygiene.”.

From May 5 to December 31, 2014, there were 5252 views of the video, mostly of the Chinese version (3509/5252, 66.81%) (see Figure 1). The production cost was US $6250, so the average cost per click during this period was US $1.20.

### Efficacy of Three Different Promotions

Among the 3509 viewers of the Chinese version, 53.01% (1860/3509) was female and 46.99% (1649/3509) male. The main age group was among 25-34 years old, which accounted for 34.0% of the viewers. We used three different Internet-based platforms to promote the Chinese version of the video. At the hospital level, the official website of NTUH had 24,000 subscribers, and 151 of them opened the link to the video (connection rate: 0.63%, 151/24,000). A total of 9967 people received the hospital group email, and 91 of them opened the link to the video (connection rate: 0.91%, 91/9967). There was no feedback from the email or website promotions. On Facebook, there were 13,080 clicked to the site, and 807 opened the link to the video. The connection rate for Facebook (6.17%, 807/13,080) was significantly higher than from the other two platforms (*P*<.001 for both comparisons). On Facebook, 525 visitors (525/13,080, 4.01%) clicked on “Like” for the HH campaign and 21 visitors (21/13,080, 0.16%) shared the video (see Table 1).

**Table 1:** Views and feedback on the hand hygiene video that were traced to different applications.

Application	Headlines (total message word counts)	Placement	Users/Visitors	Views	Interaction
NTUH Website	May 5, World Hand Hygiene Day (163)	Link	24,000	151	0
NTUH group email	May 5, World Hand Hygiene Day (163)	Link	9,967	91	0
Personal Facebook site	May 5, World Hand Hygiene Day (45)	Link	13,080	807	524 “liked”; 21 “shares”; 7 “recommends”

### Time Series Analysis

On May 5, 2014, a press release was also sent from Public Relations Office of NTUH to 20 local newspaper offices. One e-newspaper reported this message that afternoon and provided the YouTube link. On the first day, except for the direct links to the Chinese version, most viewers were from the Facebook site (n=105), followed by NTUH website (n=19) and hospital group email (n=19), and e-newspaper (n=5). One traditional newspaper reported the news on the following day.

**Figure 2:**
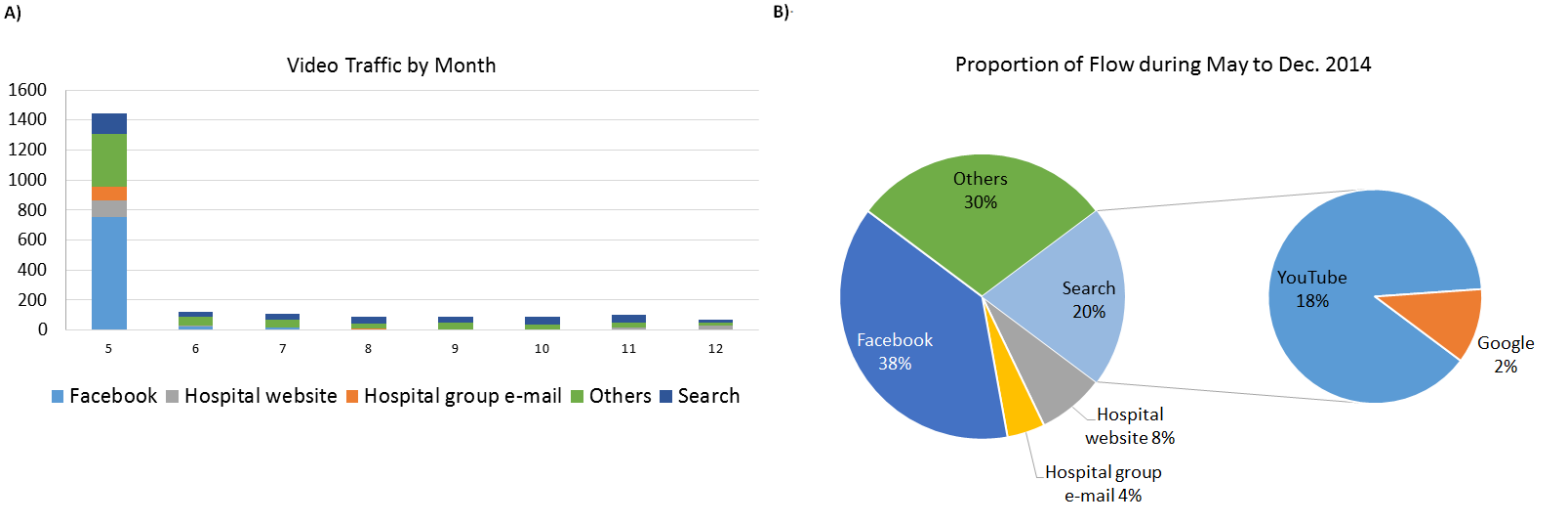
(A) The number of views of the Chinese version of the hand hygiene video from May 5 to Dec 31, 2014 (views were excluded if the source could not be traced; “Others” included YouTube recommendations, e-newspapers, and e-magazines). (B) Proportion of traffic from different sites from May 5 to Dec 31, 2014.

During the following year after the video launched (until Dec. 31, 2014), 69.68% (2445/3509) of the views of the Chinese version of the video were during the first month. After excluding 1259 direct links to the video (which cannot be readily tracked), 38.09% (857/2250) of the views were from Facebook, 7.60% (171/2250) from the NTUH website, 4.31% (97/2250) from the NTUH group email, 29.60% (666/2250) from other sources (eg, YouTube suggestions, e-newspapers, e-magazines), and 20.40% (459/2250) from search engines, including YouTube (407/2250, 18.09%) and Google (52/2250, 2.31%) (see Figure 2). The views of the video via search engines increased from 9.75% (141/1446) in May 2014 to 33.80% (24/71) in December 2014. The average time spent on the video was 2:40 minutes via the Facebook, 2:47 minutes via the NTUH, and 1:28 minutes via the group email.

### Comparison of Versions in Different Languages

**Figure 3:**
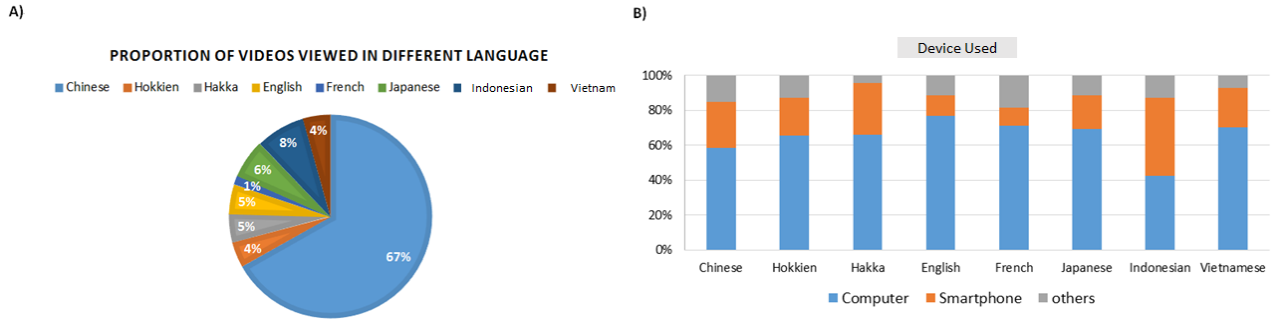
Views of the hand hygiene video in different languages and with different devices. (A) Proportion of videos viewed in each of 8 languages. (B) Devices used to view the video in each of the 8 languages.

Overall, the video was distributed in 49 countries, and on average, viewers spent 1.57 minutes on the 3-minute video. The Chinese version was the most popular (3509/5252, 66.81%), followed by the Indonesian version (414/5252, 7.88%) (see Figure 3). There were some differences in how different viewers accessed the video. For the English version, 7.4% (18/245) of the views were through Twitter and 1.2% (3/245) through Facebook.

Most visitors used computers to watch the video, except for the Indonesian version where a higher proportion was viewed by smartphone (187/414, 45.2%) than computer (174/414, 42.0%), although not statistically significant (*P*=.36) (see Figure 3).

### Hand Hygiene Compliance

The hospital-wide HH compliance improved from 83.7% (473/565) in 2013 to 86.8% (589/679) in 2014, although this change was not significant (*P*=.13) (Table 2). Among physicians, the HH compliance improved from 69.0% (69/100) to 81.1% (163/201) (*P*=.02).

**Table 2:** Hand hygiene compliance before and after the 2014 video campaign.

Compliance	2013, % (n/N)	2014, % (n/N)	*P* value
Total	83.7 (473/565)	86.7 (589/679)	.13
Physicians	69.0 (69/100)	81.1 (163/201)	.02
Nurses	87.9 (350/398)	89.9 (390/434)	.38
Others	80.6 (54/67)	81.8 (36/44)	.87

## Discussion

### Principal Findings

During the hand hygiene video campaign in 2014, the promotion on Facebook was significantly more successful than promotion on two traditional Internet-based communication tools, that is, the hospital website and group email.

Different marketing strategies have been used in the promotion of HH [9,10]; however, there is a growing influence of social media on health promotion [11]. For hand hygiene, Web-based interventions have been reported to promote hand washing during the flu season among the community [12]. In the health care setting, printed posters were traditionally used as reminders in the workplace [3]. This approach could be extended to the Internet (eg, video clips). The important issue is how to promote videos, instead of passively waiting for them to been seen. In this study, we demonstrated that social media could be a surrogate method to connect to our community of HCWs. Importantly, this new promotion tool allows quantification of the connection rate.

Through quantitative analysis, we revealed that connecting to the HH video is more efficient through Facebook than the traditional hospital website and group email. The difference in connection rate may be due to the fact that shared information among peers is an important influence on behavior [13]. The Facebook site, “Clerk: The life as a road blocker,” was run by a medical school student and followed by 13,080 users. Facebook interaction, including “Likes” and “Shares”, can create further discussion and connect to a greater possible audience. In contrast, the website and email would be more like a broadcast site without a method for further interaction.

In addition to the ability to connect to the right community and create interaction, social media also allows consumption and control, per the social media “4 Cs” (ie, connection, community, context, content) [14]. The word-of-mouth effect in social media, such as “like,” “share,” or “recommend” on Facebook, is controlled by all viewers and by the Facebook owner. We were fortunate to have a partner who received adequate infection control training and could provide professional feedback. However, other important online influencers may not have the same knowledge of infection control. Thus, there is an urgent need for the health care system to take a more proactive role in communicating with the net generation [15]. Thus, we could provide additional information about HH programs besides the video, which was designed to be short and catch viewers’ attention. However, special personnel are needed to create, maintain, and provide timely feedback on social media [16]. We hope that the current study stimulates interest in the analysis of cost and efficacy of using social media to promote infection control.

Of note, the proportion of the video viewers via Facebook decreased after the first month that the video launched. The potential pros of Facebook as rapid information sharing can also be a cons as the message may disappear rapidly. But while we stored the video campaign on the Infection Control Center’s YouTube site in December 2014, 34% of the traffic to the video was via search engine (eg, Google, YouTube). Thus, since search-engine optimization has been an important marketing tool [17], simple and frequently used keywords need to be planned early before launching a campaign.

Also from the point of view of marketing, the efficacy of a social media campaign would be considered as the return on investment (ROI). However, health care is different from commercial operations and online merchandise, so we cannot provide a traditional ROI as “cost per acquisition.” However, we calculated the equivalent cost per click as US $1.2, and this is within the average cost of online advertising [18].

There may have been some regional differences among our viewers. According to our analysis of the eight different versions of video, we found that those who viewed the Indonesian version had greater utilization of smartphones. This is consistent with the findings of a 2013 national survey in Taiwan, which found that new immigrants were more likely to own a smartphone than a computer (82.5% vs 61.4%) [19]. Thus, for future marketing of HH videos, we suggest that the material be tailored to suit the needs of different viewers and the characteristics of different electronic devices (eg, length of the video, formatting).

### Limitations

A limitation of our study would be that HH behavior may be influenced by other interventions at the same time. In Taiwan, the national HH program was promoted by CDC, Taiwan, in 2012. There were no other concurrent interventions for HH after that within the institution. The HH compliance was also measured by professional ICNs, and objective compliance rate was used for evaluation.

Another limitation of this study is that we did not examine the use of multiple social media platforms. In particular, different geographical regions tend to prefer different social media sites, as is the case with Facebook and Myspace [20]. Surprisingly, the video had been distributed to 49 countries. Even though this effect may beyond the scope of this study, we are aware that video campaigns via novel social media can reach even larger populations beyond geographic boundaries. As Twitter had been used more frequently than Facebook in our English version, other social media platforms could also be considered for marketing an HH program, such as Twitter, blogs, mini-blogs, Instagram, Tumblr, Google^+^, and Vimeo. Future research could focus on regional differences in the use of different social media websites.

### Conclusions

Bernoff et al stated that social media “is a phenomenon of people connecting with each other and drawing strength from each other” [21]. This echoes the long-term goal of the HH program to empower our patients and create a safe environment in health care facilities. We expect that use of novel social media websites may play useful roles in the promotion of HH programs. Thus, starting a blog, building an online infection control community, or creating a dedicated Facebook page will allow us to engage more people and increase HH awareness.

## Abbreviations

CDC: Centers for Disease Control (Taiwan)
HCWs: health care workers
HH: hand hygiene
NTU: National Taiwan University
NTUH: National Taiwan University Hospital
